# Identification and Characterization of a Novel Plasmid-Encoded Laccase-Like Multicopper Oxidase from *Ochrobactrum* sp. BF15 Isolated from an On-Farm Bio-Purification System

**DOI:** 10.17113/ftb.59.04.21.7253

**Published:** 2021-12

**Authors:** María Carla Martini, Francesca Berini, Luka Ausec, Carmine Casciello, Carolina Vacca, Mariano Pistorio, Antonio Lagares, Ines Mandic-Mulec, Flavia Marinelli, María Florencia Del Papa

**Affiliations:** 1 IBBM - Institute of Biotechnology and Molecular Biology, CONICET - Department of Biological Sciences, Faculty of Exact Sciences, National University of La Plata, Calles 47 y 115 (1900) La Plata, Argentina; 2 Department of Biotechnology and Life Sciences, University of Insubria, via J.H. Dunant 3, 21100 Varese, Italy; 3 Department of Food Science and Technology, Biotechnical Faculty, University of Ljubljana, Večna pot 111, 1000 Ljubljana, Slovenia

**Keywords:** laccase-like multicopper oxidases, *Ochrobactrum*, biopurification system, plasmid, biodegradation, heterologous expression

## Abstract

**Research background:**

In recent decades, laccases (*p*-diphenol-dioxygen oxidoreductases; EC 1.10.3.2) have attracted the attention of researchers due to their wide range of biotechnological and industrial applications. Laccases can oxidize a variety of organic and inorganic compounds, making them suitable as biocatalysts in biotechnological processes. Even though the most traditionally used laccases in the industry are of fungal origin, bacterial laccases have shown an enormous potential given their ability to act on several substrates and in multiple conditions. The present study aims to characterize a plasmid-encoded laccase-like multicopper oxidase (LMCO) from *Ochrobactrum* sp. BF15, a bacterial strain previously isolated from polluted soil.

**Experimental approach:**

We used *in silico* profile hidden Markov models to identify novel laccase-like genes in *Ochrobactrum* sp. BF15. For laccase characterization, we performed heterologous expression in *Escherichia coli*, purification and activity measurement on typical laccase substrates.

**Results and conclusions:**

Profile hidden Markov models allowed us to identify a novel LMCO, named Lac80. *In silico* analysis of Lac80 revealed the presence of three conserved copper oxidase domains characteristic of three-domain laccases. We successfully expressed Lac80 heterologously in *E. coli*, allowing us to purify the protein for further activity evaluation. Of thirteen typical laccase substrates tested, Lac80 showed lower activity on 2,2'-azino-bis(3-ethylbenzothiazoline-6-sulphonic acid) (ABTS), pyrocatechol, pyrogallol and vanillic acid, and higher activity on 2,6-dimethoxyphenol.

**Novelty and scientific contribution:**

Our results show Lac80 as a promising laccase for use in industrial applications. The present work shows the relevance of bacterial laccases and highlights the importance of environmental plasmids as valuable sources of new genes encoding enzymes with potential use in biotechnological processes.

## INTRODUCTION

Enzymes traditionally known as laccases are multicopper oxidases that catalyse the oxidation of a wide range of substrates and the simultaneous reduction of molecular oxygen to water. Due to their ability to oxidize a wide variety of phenolic and non-phenolic compounds, they have been extensively used in biotechnological processes as biocatalysts (*1*, *2*). They were recently renamed ’laccase-like multicopper oxidases’ (LMCOs), following the nomenclature revision of this enormously diverse group of enzymes (*3*). LMCOs are produced by a wide range of organisms such as bacteria, higher plants, insects and fungi, the fungi being the more broadly studied and characterized, in part due to the development of efficient expression systems in yeasts that have potentiated their industrial use, particularly in the textile and food industry (*4*-*7*). However, much attention has been given more recently to prokaryote-sourced LMCOs. Bacterial laccases usually display higher thermal and alkaline pH stability than their eukaryotic counterparts and are active on a broad range of substrates, unlike most fungal laccases (*3*, *8*), making them suitable for different industrial processes. A computational study analyzing over 2000 bacterial genomes predicted the presence of laccase-like encoding genes in 36% of the screened organisms (*9*) including autotrophic, alkalophilic and even anaerobic bacteria (*10*), highlighting the extraordinary potential of bacteria as a source of novel LMCOs and pointing out the need to continue exploring the genome of these microorganisms.

Multiple approaches have been applied for the identification of novel LMCOs in bacteria, including classical functional- and/or sequence-based screenings of culturable microbiota (*11*), or metagenomic analyses (*10*, *12*). In addition, the generation of chimeric laccases using bioinformatic approaches has also generated new hybrid enzymes with promising activity (*13*). Interestingly, the use of bacterial consortia has been proved to be a good alternative for screening of multicopper oxidase activity (*14*, *15*).

Despite the increasing interest in bacterial LMCOs, little attention has been paid to plasmid-encoded laccases. We previously published a bioinformatic analysis in which we analyzed the presence of laccase-encoding genes in a variety of bacterial genomes from different species (*9*). We found that, of the 749 genes identified in finished genomes, 10% were encoded on plasmids; 68% of these genes codify three-domain laccases and 32% two-domain laccases. These findings highlight the importance of plasmids – and other mobile genetic elements (MGE) – as reservoirs of laccases and other enzymes.

In an attempt to identify novel laccases for potential use in industry or bioremediation, we screened a plasmid metagenome obtained from over 50 plasmids purified from bacterial strains carrying high-molecular mass plasmids. This bacterial collection consists of 35 organisms that include both Gram-positive and Gram-negative bacteria belonging to 14 genera, all of them isolated from a biopurification system used for the remediation of pesticide-contaminated waters in Kortrijk, Belgium (*16*). Exposure of these indigenous bacteria to mixtures of pollutants has fostered their adaptation responses *via* horizontally acquired MGE. Of the MGE, plasmids are the most abundant and promiscuous ones, representing the main vehicles for horizontal gene transfer *via* conjugation in bacterial communities in polluted environments (*17*, *18*).

Considering the relatively great abundance of laccase-encoding genes in plasmids predicted *in silico* (*9*), we hypothesized that the plasmids from the 35 strains are a possible source of laccases. To identify novel LMCOs, we computationally screened this plasmid data set. We were able to identify one full-length gene for a putatively novel LMCO, herein named *lac80*. The heterologous expression of the codon-optimized version of *lac80* in *Escherichia coli* followed by His-Tag purification allowed us to assess its activity on different substrates, exploring its potential use in bioprocesses.

## MATERIALS AND METHODS

### Strains and vectors used in this study

Bacterial isolates used in this study were previously obtained from a biopurification system used for pesticide removal from contaminated waters located in Kortrijk, Belgium, operational since 2008 (*16*). All strains were routinely grown on Luria-Bertani (LB) agar plates or in liquid LB medium (Sigma–Aldrich, Merck, St Louis, MO, USA) at 37 °C and 200 rpm. The biopurification system composition, analysis of pesticide types and concentrations (*19*), high molecular mass (HMM) plasmid DNA purification, high-throughput sequencing and computational analysis were previously reported (*20*). *Escherichia coli* DH5α (Promega, Madison, WI, USA) and *E. coli* BL21 Star^TM^ (DE3) (Invitrogen Life Technologies, Carlsbad, CA, USA) strains were employed for *lac80* cloning and heterologous expression, respectively. They were routinely cultured in LB medium supplemented when appropriate with 100 μg/mL of ampicillin (Sigma–Aldrich, Merck). The pUC59 (GenScript Biotech, Piscataway, NJ, USA) and pET22b(+) (Novagen, Darmstadt, Germany) plasmids were used for *lac80* cloning and expression, respectively. The latter plasmid enabled us to introduce a hexahistidine tag (His_6_-Tag) at the C-terminus of the protein. Molecular biology techniques were performed using the standard protocols (*21*). All the reagents were purchased from Sigma–Aldrich, Merck unless otherwise stated.

### Screening and lac80 sequence analysis

Laccase-like multicopper oxidase (LMCO)-encoding *lac80* gene was identified *in silico* in the present study from the plasmid dataset reported by Martini *et al.* (*20*). To retrieve novel LMCOs, profile hidden Markov models (pHMMs) were used as previously described (*9*). The *lac80* gene and aminoacidic sequences were compared to those reported in GenBank (*22*) and Protein Data Bank (*23*), respectively. The protein module structure was analyzed using the simple modular architecture research tool (SMART) (*24*). The presence and location of the signal peptide in *lac80* was checked using the neural networks and hidden Markov models trained on Gram-negative and Gram-positive bacteria with SignalP 5.0 (*25*). Multiple sequence alignment of *lac80* with related LMCO sequences was performed using ClustalΩ (*26*). Laccase gene was also blasted to the Laccase Engineering Database (*27*) by the basic local alignment search tool (BLASTP) algorithm (*28*). Phylogenetic relationship was inferred by using the maximum likelihood phylogenetic method. The tree was constructed using MEGA X (*29*) and a multiple sequence alignment by MUSCLE of Lac80 sequence with a selection of previously characterized and some uncharacterized bacterial LMCOs, and 100 bootstrapping replications were used as a test of phylogeny.

A molecular polymerase chain reaction (PCR)-based method was used to screen the bacterial collection for retrieving this novel LMCO-encoding gene. The strain harbouring *lac80* was identified by using two sets of primers (set 1: 5’-CCACCGTCTGGGGTCTTG-3’ and 5’-GTCGATGCGCCGTATTTC-3’, amplicon size 549 bp and set 2: 5’-TCACCGGGCGATGCTGGC-3’ and 5’-GAGGAGGTGATGGCCGAGATC-3’, amplicon size 733 bp). PCR was performed as follows: an initial denaturation step at 94 °C for 4 min, followed by 35 cycles of denaturation at 94 °C for 20 s, annealing at 54 °C for 30 s and extension at 72 °C for 50 s. Then, a final extension at 72 °C for 2 min was used. The sequence of *lac80* was submitted to GenBank (*22*) under the accession number MT130716.

### Genetic localization of lac80

*In situ* lysis gel electrophoresis was performed to obtain further information on the genomic location of *lac80* by analyzing the DNA of the bands corresponding to plasmid(s) or chromosome, as previously described (*16*). The visualized bands were purified using the AccuPrep PCR/gel purification kit (Bioneer, Daejeon, Korea). Then, a semi-nested PCR for *lac80* was run to reduce nonspecific amplification of DNA template. Purified samples and primers Lac80F1 (5’-CCACCGTCTGGGGTCTTG-3’) and Lac80R2 (5’-TCACCGGGCGATGCTGGC-3’) were used in the first PCR and Lac80F1 and Lac80R1 (5’-GTCGATGCGCCGTATTTC-3’) in the second PCR run. To exclude the amplification of contaminating DNA, several controls were included in the PCR with DNA recovered from randomly selected positions of the agarose gel.

### Codon optimization

The codon optimization strategy employed in the present study is referred to as ‘one amino acid-one codon’. In this method, the most preferred codon of the *E. coli* expression system for a given amino acid is utilized in the target sequence (*30*). The sequence of *lac80* was obtained from the data set. The OPTIMIZER web server (*31*) was used for rare codon detection. The *E. coli* rare codon analyzer2 (*32*) was utilized for gene sequence optimization. GenScript web server (*33*) was used to analyze the designed sequence codon adaptation index (CAI).

### Cloning and heterologous expression of lac80 in E. coli

The codon-optimized sequence of *lac80* with the native signal peptide removed was synthesized by GenScript Biotech (Piscataway, NJ, USA) and cloned into pUC59 plasmid (Leiden, The Netherlands) for DNA amplification. For protein expression, *lac80* was cloned from pUC59 into the expression vector pET22b(+) (Novagen Inc, Madison, WI, USA), and introduced into *E. coli* BL21 Star^TM^ (DE3) cells (Thermo Fisher Scientific, Waltham, MA, USA). Multiple conditions were then evaluated for optimizing Lac80 expression. Briefly, 2 L unbaffled Erlenmeyer flasks containing 750 mL of LB medium (Merck KGaA, Darmstadt, Germany) or richer Terrific broth (TB, in g/L: tryptone 12, yeast extract 24, K_2_HPO_4_/L 9.4, KH_2_PO_4_ 2.2, glycerol 8; components from Merck KGaA) supplemented with ampicillin 100 µg/mL (Merck KGaA) were inoculated with the starter culture (*A*_600 nm_~0.1), then incubated at 37 °C and 200 rpm. When the early exponential phase of growth was reached (corresponding to an *A*_600 nm_ of approx. 0.6 for LB medium and approx. 1 for TB medium), different concentrations of isopropyl-β-d-thiogalactopyranoside (IPTG, from 0.4 to 1 mM) (Merck KGaA) were tested to induce protein expression. At the same time, none or 0.25 mM CuSO_4_ (Merck KGaA) was added to facilitate metal incorporation into the enzyme active site. Cells were then incubated at 37 or 20 °C under different agitation regimes (200 or 100 rpm or without shaking), and harvested at 0, 2, 4 or 24 h after induction, by centrifuging at 7500×*g* for 10 min (Thermo Fisher Scientific, Langenselbold, Germany). Multiple combinations of incubation conditions were also evaluated, such as incubation for 4 h after induction at 37 or 20 °C and 200 rpm, followed by overnight incubation at the same temperature but without shaking. Total proteins in the cell-free fermentation broths were concentrated by precipitation with *φ*(trichloroacetic acid)=10%. Cell pellets were instead resuspended in 20 mM phosphate buffer, pH=6.7, containing 10 µg/mL deoxyribonuclease I (DNaseI) (Merck KGaA), 0.19 mg/mL phenylmethylsulfonylfluoride (PMSF) and 0.7 µg/mL pepstatin (Merck KGaA). Then, cells were disrupted by sonication for 6 cycles of 30 s each on ice, followed by centrifugation at 34 000×*g* for 60 min at 4 °C (Beckman Coulter Inc., Brea, CA, USA). This enabled us to recover soluble (cytoplasmic) and insoluble (inclusion bodies) fractions, which were then analyzed through: (*i*) sodium dodecyl sulfate-polyacrylamide (12% *m*/*V*) gel electrophoresis (SDS-PAGE) and Coomassie Brilliant Blue staining, (*ii*) Western blot with anti-His-Tag antibody horseradish peroxidase (HRP) conjugate (Novagen Inc., Madison) and detection by chemiluminescence (ECL Western blotting detection system, GE Healthcare Sciences, Little Chalfont, UK), and (*iii*) routine laccase activity assay with 2,2'-azino-bis(3-ethylbenzothiazoline-6-sulphonic acid) (ABTS) (Merck KGaA) as substrate (see below). Total protein concentration in the fractions was estimated by the biuret assay (*34*).

### Lac80 purification

Lac80 was purified starting from recombinant *E. coli* cells grown under the conditions allowing the highest soluble protein production. Hence, glycerol stocks of *E. coli* BL21 Star^TM^ (DE3)/pET22(b)::*lac80* were inoculated into 80 mL LB medium with 100 µg/mL ampicillin, grown overnight at 37 °C and 200 rpm. This pre-culture was then used to inoculate (at *A*_600 nm_~0.1) 750 mL of selective LB medium. Flasks were incubated as before until an *A*_600 nm_ of 0.6 was reached. After induction of protein expression with 0.4 mM isopropyl β-d-1-thiogalactopyranoside (IPTG) and addition of 0.25 mM CuSO_4_, incubation was prolonged overnight at 20 °C and 100 rpm. Cells were disrupted as reported above and the soluble, cytoplasmic fraction recovered by centrifugation at 34 000×*g* and 4 °C for 60 min (Beckman Coulter Inc.). C-terminus His_6_-tagged Lac80 was purified by affinity chromatography on a 5-mL Ni^2+^-HiTrap chelating affinity column (GE Healthcare Sciences) equilibrated with 20 mM phosphate buffer, pH=6.7, 20 mM imidazole and 300 mM Na_2_SO_4_ (Merck KGaA). The recombinant protein was eluted with 20 mM phosphate buffer, pH=6.7, 250 mM imidazole and 300 mM Na_2_SO_4_, then loaded onto a size-exclusion PD10 Sephadex G25 column (GE Healthcare Sciences) equilibrated with 20 mM phosphate buffer, pH=6.7. Lac80 was further concentrated with 30 K Amicon Ultra-2 centrifugal filter devices (Merck KGaA).

Protein purity was checked with 10% *m/V* SDS-PAGE. For molecular mass determination, molecular mass markers (GE Healthcare Sciences) were used. Protein concentration was estimated by densitometric analysis of SDS-PAGE bands using Quantity One analysis software (*35*) (Bio Rad, Hercules, USA) and spectrophotometrically using Lac80 theoretical molar absorption coefficient at 280 nm calculated based on the amino acid sequence (*ε*_280 nm_=58.44 mM^-1^cm^-1^).

### Enzymatic activity of Lac80

Laccase activity was routinely measured at 25 °C in 1 mL reaction mixture containing 50 mM sodium acetate, pH=5.0, 5 mM ABTS (*ε*_420 nm_=36 mM^-1^cm^-1^) and 100 μL protein sample. Oxidation of the substrate was monitored with a spectrophotometer (V460; Jasco, Easton, MD, USA) as the change in the absorbance at 420 nm for 5 min. All assays were conducted in triplicate and negative controls without enzyme were run in parallel. One activity unit (U) was defined as the amount of Lac80 required to oxidize 1 μmol of ABTS per minute. The ability of Lac80 to oxidize various phenolic and non-phenolic compounds was checked by the following additional substrates at designed concentrations and wavelengths: 100 mM 2,6-dimethylphenol (DMP) (*ε*_468 nm_=49.6 mM^-1 ^cm^-1^), 100 mM pyrocatechol (*ε*_450 nm_=2.21 mM^-1^cm^-1^), 100 mM pyrogallol (*ε*_450 nm_=4.4 mM^-1^cm^-1^), 8 mM vanillic acid (*ε*_316 nm_=2.34 mM^-1^cm^-1^), 10 mM K_4_Fe(CN)_6_ (*ε*_405 nm_=0.9 mM^-1^cm^-1^), 2 mM tyrosine (*ε*_475 nm_=3.6 mM^-1^cm^-1^), 5 mM l-3,4-dihydroxyphenylalanine (l-DOPA) (*ε*_475 nm_=3.7 mM^-1^cm^-1^), 100 mM guaiacol (*ε*_468 nm_=12 mM^-1^cm^-1^), 10 mM syringic acid (*ε*_300 nm_=8.5 mM^-1^cm^-1^), 4 mM ferulic acid (*ε*_287 nm_=12.4 mM^-1^cm^-1^), 1 mM syringaldazine (*ε*_525 nm_=65 mM^-1^cm^-1^) and 1 mM syringaldehyde (*ε*_320 nm_=8.5 mM^-1^cm^-1^). The optimal pH for the activity on different substrates was evaluated at 25 °C in a multi-component buffer (10 mM Trizma base, 15 mM sodium carbonate, 15 mM phosphoric acid, 250 mM potassium chloride (*36*), in the pH range 2.0–8.0. All substrates and buffer components were purchased from Merck KGaA.

## RESULTS AND DISCUSSION

### Lac80 is a plasmid-encoded three-domain laccase

**Fig. 1:**
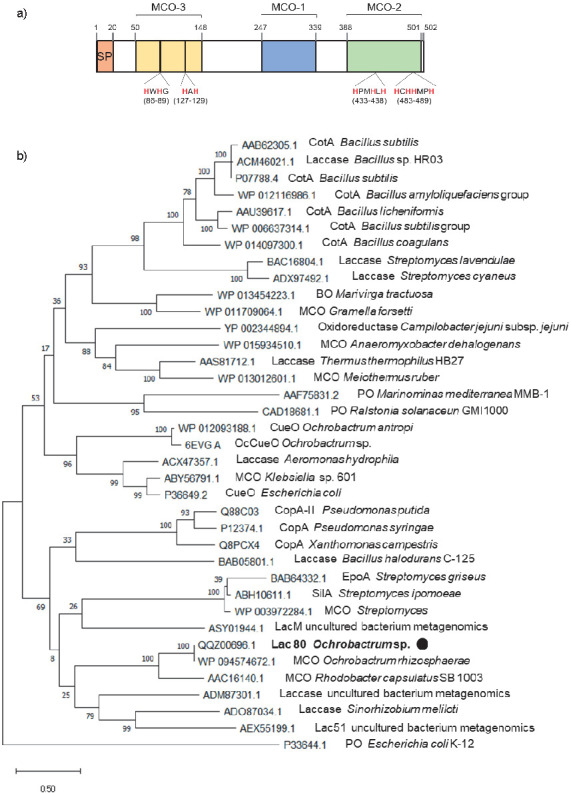
*In silico* analysis of Lac80 protein sequence: a) predicted signal peptide (SP), oxidase type 3 domain (MCO-3) PF07732, oxidase type 1 domain (MCO-1) PF00394 and oxidase type 2 domain (MCO-2) PF07731 are indicated. Residues involved in the coordination of the copper atoms are highlighted in red, and b) maximum likelihood phylogenetic tree of *Ochrobactrum* sp. BF15 putative laccase-like multicopper oxidase (LMCO) based on reference protein sequences of characterized and some uncharacterized bacterial laccases from Protein Databases (*22*, *23*). The percentage of trees in which the associated taxa cluster together is shown next to the branches. MCO=multicopper oxidase, BO=bilirubin oxidase, PO=polyphenol oxidase. Solid black circle indicates the protein characterized in this study

Polluted sites are hot spots of plasmids potentially carrying catabolic genes (*37*). To identify novel laccase-like multicopper oxidase (LMCO)-encoding genes in the plasmid dataset from the 35 strains previously isolated from a biopurification system [REMOVED HYPERLINK FIELD] (*20*), a profile hidden Markov model-based search was performed. One candidate gene, named *lac80*, was retrieved using this approach. This gene showed 89% identity at nucleotide level with a yet uncharacterized multicopper oxidase from *Ochrobactrum anthropi* strain OAB (GenBank accession number: CP008819). Lac80 is composed of 502 amino acids, with a predicted signal peptide of 20 amino acids at the N-terminal (Fig. 1a). Predicted isoelectric point and molecular mass of this protein, excluding the signal peptide, were 5.43 and 51.2 kDa, respectively. As reported in Table S1, full-length Lac80 protein showed the highest amino acid sequence identity with two putative multicopper oxidases, one from *Alphaproteobacteria* (WP_024899901.1, 100% sequence identity) and the other from *Ochrobactrum rhizosphaerae* (WP_094574672.1, 99.8%). In addition, Lac80 showed 30% sequence identity with the Lac15 from a marine microbial metagenome (Protein Data Bank (PDB): 4F7K_A) and 29.5% identity with a laccase from *Pseudomonas thermotolerans* (PDB: 6VOW). These two latter proteins were crystallized and characterized as functional LMCOs. The phylogenetic tree shown in Fig. 1b confirms the novelty of our enzyme since Lac80 clusters most closely with uncharacterized LMCOs from *Paracoccus* sp., *Ochrobactrum* sp. and *Rhizobiales*. These findings were also confirmed by the best protein matches identified by the Laccase Engineering Database (LccED, https://lcced.biocatnet.de/) (*27*) by the basic local alignment search tool (BLASTP) algorithm for laccase-like protein.

**Fig. S1:**
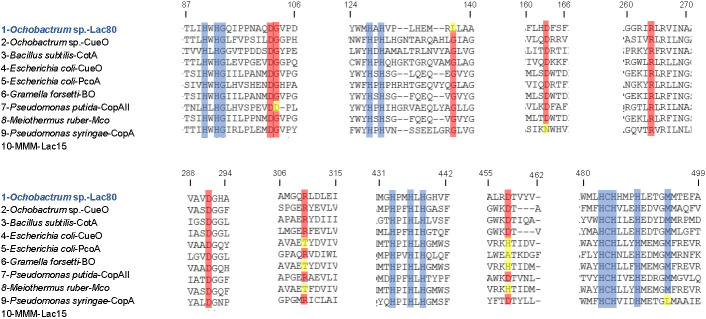
Partial amino acid multiple sequence alignment, showing 10 bacterial laccase-like multicopper oxidases. Metal-binding residues are coloured in blue; other conserved residues are highlighted in red, whereas mismatches are in yellow. Numbering is based on Lac80 sequence as reference. Protein data Bank (23) or GenBank (22) accession codes are as follows: 1=QQZ00696.1, 2=6EVG|A, 3=P07788, 4=P36649, 5=A0A0E0XT94, 6=(GB) WP_011709064.1, 7=Q88C03, 8=(GB) WP_013012601.1, 9=P12374, 10=E1ACR6. BO=bilirubin oxidase, MMM=marine microbial metagenome.

*In silico* sequence analysis of Lac80 identified the three conserved copper-oxidase domains characteristic of laccases, with protein families (Pfam) database accession numbers of PF07732, PF00394 and PF07731 (Fig. 1a). Multiple sequence alignment with bacterial laccases or multicopper oxidase sequences reported in Fig. S1 revealed that Lac80 contains metal-binding amino acid residues that are conserved in bacterial laccases.

**Fig. 2:**
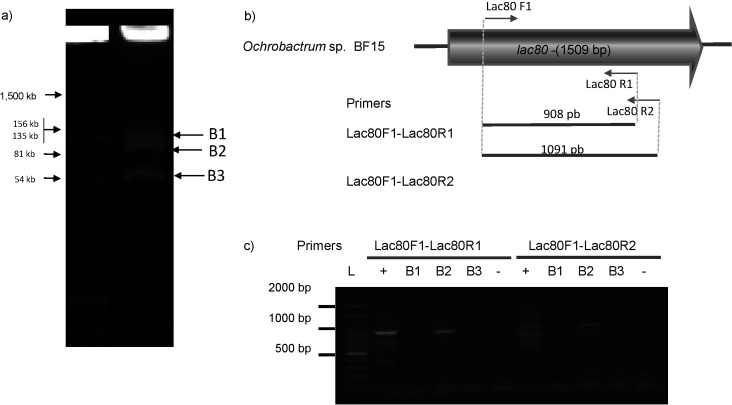
Localization of *lac80* in *Ochrobactrum* sp. BF15: a) *in situ* gel electrophoresis showing plasmid profile (line 2). The well-known plasmid profile of *Sinorhizobium meliloti* MVII-1 (line 1) (*32*) was used to estimate the size of *Ochrobactrum* sp. BF15 plasmids, b) schematic localization of primers used for *lac80* plasmid localization and expected PCR products, and c) agarose gel showing the PCR products obtained with total DNA from *Ochrobactrum* sp. BF15 or from different plasmids (B1, B2 and B3) purified from the bands. +=positive control, −=negative control, L=ladder

To identify the strain containing *lac80*, a PCR-based screening (see Materials and Methods) was performed using gDNA from the 35 strains (*38*). Of all the tested strains, only one yielded positive PCR results. Identification of the PCR product was corroborated by sequencing. The strain carrying the plasmid-encoded *lac80* gene was identified as *Ochrobactrum* sp. BF15. To determine the genomic localization of *lac80*, an additional PCR screening was performed using the DNA bands cut from the *in situ* lysis gel electrophoresis (*16*) (Fig. 2a and Fig. 2b). Positive amplification from plasmid bands (Fig. 2c) and further sequencing confirmed that *lac80* is encoded in a plasmid.

**Fig. S2:**
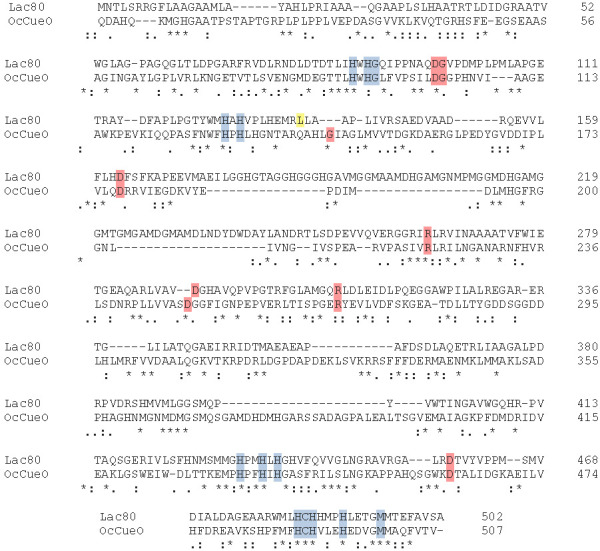
Sequence alignment of Lac80 with OcCueO from *Ochrobactrum* sp. Alignment was carried out using Clustal Omega online tool (*26*). The percentage of identity is 25.5%. Metal-binding residues are coloured in blue; other conserved residues among the bacterial laccase-like multicopper oxidases shown in Fig. S1 are highlighted in red, whereas mismatches are in yellow. Uniprot (*23*) or GenBank (*22*) accession codes as follows: Lac80 (QQZ00696.1), OcCueO (6EVG|A)

Although laccases have already been found in *Ochrobactrum* spp. (*39*-*41*), this is the first report of a plasmid-encoded LMCO in this genus. *Ochrobactrum* species are aerobic, non-fermenting, Gram-negative bacilli found in a variety of environmental sources such as water, soil, plants and animals. Genome sequencing of *Ochrobactrum* strains has shown the presence of multiple circular chromosomes and large plasmids (*42*-*46*). In addition, plasmid conjugation machinery has been identified in *Ochrobactrum* plasmids (*20*, *42*), and horizontal gene transfer by conjugation was demonstrated in this genus (*47*, *48*). *Ochrobactrum* species was previously found to degrade a wide spectrum of recalcitrant and xenobiotic compounds, such as organophosphates (*49*), tetrachloroethene (*50*), dichloro-diphenyl-trichloroethane (DDT) (*51*), 2,4-dichlorophenoxyacetic acid, endosulfan (*52*) and aniline (*53*), among many others. Interestingly, ligninolytic activity was also reported from *Ochrobactrum* species (*39*, *41*). However, this is the first report of a plasmid-encoded functional laccase in the *Ochrobactrum* genus. Granja-Travez *et al.* (*39*) reported the purification and characterization of a laccase, named OcCueO, from *Ochrobactrum* sp. Although this laccase has the typical three-domain multicopper oxidase structure and displays activity against different substrates, alignment of OcCueO with Lac80 showed a low percentage of identity at the amino acid level (25.75%) (Fig. S2). This is not surprising, considering that OcCueO appears to be encoded in the chromosome, whereas l*ac80* is located in a plasmid, indicating a possible acquisition *via* horizontal gene transfer and consequently different evolution pathways.

### Heterologous expression and purification of Lac80

**Fig. 3:**
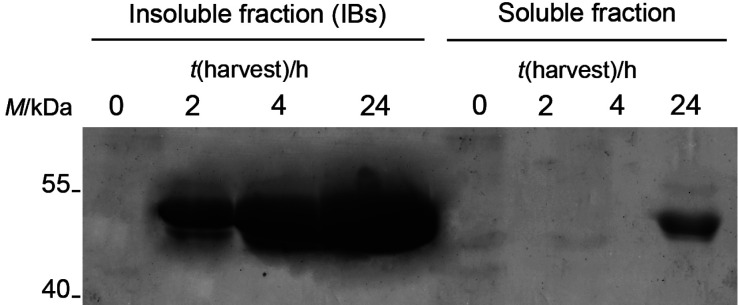
Heterologous expression of Lac80. Western blot analysis of soluble and insoluble fractions from *E. coli* BL21 Star^TM^ (DE3)/pET22(b)::*lac80* cells was performed. Cells were grown in LB medium, supplemented with 0.25 mM CuSO_4_ at the same time of IPTG induction, then incubated at 20 °C and 100 rpm and harvested 0, 2, 4 or 24 h after induction. The loaded samples correspond to the soluble or insoluble fractions from cells recovered from *V*(culture)=1 or 5 mL, respectively. IB=inclusion bodies

The putative LMCO gene was synthesized by optimizing its codon usage for *E. coli* expression and removing the signal peptide sequence. The synthetic gene was then cloned into the pET22b(+) plasmid, and expressed as a His_6_-tagged protein in *E. coli* BL21 Star^TM^ (DE3). Different expression conditions were explored, as detailed in Materials and Methods. Briefly, recombinant cells were grown in two dissimilarly composed cultivation media and increasing concentrations of IPTG were tested to induce Lac80 expression. Following IPTG addition, incubation under diverse temperatures and shaking regimes was compared, together with the addition of CuSO_4_. Under most of the above conditions, SDS-PAGE and Western blot analyses revealed that His_6_-Lac80 (molecular mass of 52.5 kDa) accumulated in the inclusion bodies (IBs) in an insoluble form. The recombinant protein was never detected in the extracellular broth, indicating that it could not be secreted. Laccase activity assays that used ABTS as substrate proved that Lac80 accumulated in the IBs was completely inactive, as generally expected for recombinant proteins packed into IBs in *E. coli* (*12*, *54*). This is not surprising, as other LMCOs expressed in *E. coli* were described to accumulate as inactive protein into IBs. As previously reported, the addition of copper under microaerobic conditions was used to facilitate proper protein folding, a condition that facilitates copper incorporation into the LMCO active sites (*55*). Thus we grew *E. coli* BL21 Star^TM^ (DE3)/pET22(b)::*lac80* cells in LB medium, supplemented with 0.25 mM CuSO_4_ at the moment of induction, and incubated overnight at 20 °C and 100 rpm or without shaking. This procedure allowed us to obtain traces of active recombinant Lac80 in the soluble cytoplasmic fraction detectable by both Western blot analysis and activity assay (Fig. 3). Although under these conditions most of the recombinant enzyme was still packed into IBs, approx. 50 µg of soluble Lac80 per L corresponding to approx. 10.6 µg per g cells was produced in the cytoplasmatic fraction. Activity assay (see below) confirmed that this soluble Lac80 was biologically active. Therefore, it was purified by HisTag affinity chromatography, following the procedure described in Materials and Methods. As a precaution, we prepared all the buffers with Na_2_SO_4_ rather than with NaCl, since chlorine ions were shown to inhibit laccase activity (*8*). Approx. 42 µg per L culture and 9 μg per g cells of highly pure Lac80 were recovered from the elution peak, with a purification yield of approx. 84%.

### Lac80 displays activity on different substrates

**Fig. 4:**
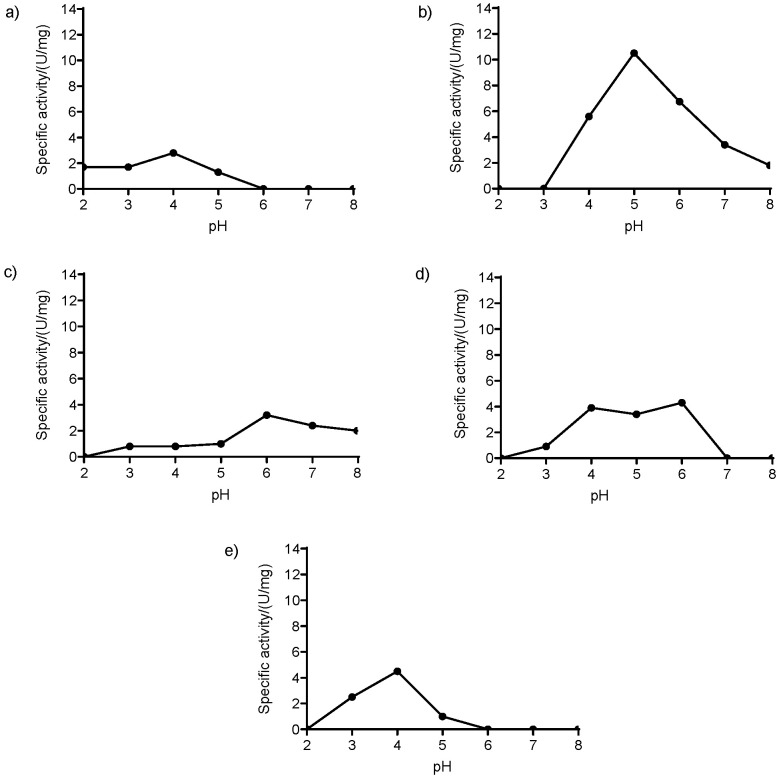
Enzymatic activity of Lac80 under different conditions. Lac80 displayed activity against: a) ABTS, b) DMP, c) pyrocatechol, d) pyrogallol, and e) vanillic acid at 25 °C and in a multi-component buffer in the pH range from 2.0 to 8.0, with 50 μM CuSO_4_ supplemented to the assay mixture. Values represent mean±standard error of three parallel measurements; when not shown, the error is smaller than the symbol used

We tested the ability of Lac80 to oxidize different phenolic and non-phenolic compounds that are typical LMCO substrates. Initial substrate screening was conducted at pH=5.0. Lac80 was active on five of the thirteen screened substrates: the non-phenolic ABTS (activity=1.25 U/mg) and the phenolic 2,6-DMP (activity=10.4 U/mg), pyrocatechol (activity=0.85 U/mg), pyrogallol (activity=3.2 U/mg) and vanillic acid (activity=0.87 U/mg). The highest specific activity at pH=5.0 was reported using 2,6-DMP as substrate, whereas oxidation of the other substrates was overall less efficient. Since for oxidation with LMCOs the optimal pH can vary among substrates, the activity of Lac80 on the five positive substrates was further evaluated at different pH values (from 2.0 to 8.0), as shown in Fig. 4. In all cases, the pH-dependent activity showed a bell-shaped curve, with optimum activity in the acidic range. These results show that Lac80 was active in a wide pH range: on 2,6-DMP, for instance, the recombinant LMCO was active in the pH interval from 4.0 to 8.0, whereas on pyrocatechol Lac80 showed activity from pH=3.0 to 8.0. Notably, on this latter substrate, approx. 65 and 53% of activity was also maintained at pH=7.0 and 8.0, respectively. A review of the literature shows that most of the thirteen substrates we tested with Lac80 were indeed oxidized by bacterial laccases in more than half of the reported cases (*56*, *57*). Unlike other LMCOs, Lac80 was inactive on tyrosine, thereby excluding the possibility of being a tyrosinase.

Interestingly, the recently characterized laccase OcCueO from *Ochrobactrum* sp. showed activity against ABTS and DMP, two substrates that Lac80 was able to degrade (*39*). However, OcCueO displayed activity against guaiacol, while Lac80 showed no activity against this substrate. Given the differences at the amino acid level, these results are expectable. It is important to highlight here the prominence of *Ochrobactrum* species as a source of phenol oxidases.

## CONCLUSIONS

Although purification of Lac80 yielded small amounts of active protein, we were able to characterize this laccase using typical substrates. Lac80 shows promising traits such as substrate flexibility and stability at different pH values, making it suitable for industrial purposes. Our further investigations will explore innovative platforms of heterologous expression coupled with using different constructs to overcome the limitations of *E. coli* system for producing Lac80. In subsequent trials, we will also test other soil-dwelling prokaryotic microbes such as bacilli or streptomycetes, known for their ability to secrete recombinant proteins, to facilitate Lac80 recovery and increase its purification yield. More comprehensive assessment of the biotechnological potential of Lac80 will be possible once the issue of its supply has been solved.

Finally, our study highlights the power of massive sequencing techniques for the discovery of novel oxidizing enzymes in still poorly investigated bacterial genera, especially those encoded in mobile genetic elements. We show that *Ochrobactrum* species are a promising source of laccase-like enzymes. A still-open question is the physiological function and ecological role of this plasmid-carried laccase-like multicopper oxidase and the way it contributes to the degradation capabilities of soil microbial communities exposed to pollution.

## Figures and Tables

**Table S1:** BLASTP alignment of the putative laccase-like multicopper oxidase protein sequence (Lac80) encoded by a plasmid-carried gene from Ochrobactrum sp. BF15. The ten best protein matches identified in NCBI by BLASTP (28) algorithm for Lac80 protein are shown

Accession	Description	Protein length		Organism	Identity/%	E value	Max score	Total score	Query cover/%
WP_024899901.1	multicopper oxidase domain-containing protein		502	*Alphaproteobacteria*	100.00	0	1000	1000	100.00
WP_094574672.1	multicopper oxidase domain-containing protein		502	*Ochrobactrum rhizosphaerae*	99.80	0	999	999	100.00
WP_138787545.1	multicopper oxidase domain-containing protein		502	*Ochrobactrum haematophilum*	99.80	0	999	999	100.00
WP_036709831.1	multicopper oxidase domain-containing protein		502	*Paracoccus sanguinis*	99.60	0	997	997	100.00
WP_155044758.1	multicopper oxidase domain-containing protein		502	unclassified *Paracoccus*	98.80	0	993	993	100.00
WP_085378474.1	multicopper oxidase domain-containing protein		493	*Paracoccus contaminans*	91.83	0	877	877	100.00
WP_126156115.1	multicopper oxidase domain-containing protein		493	*Paracoccus haematequi*	91.63	0	873	873	100.00
WP_121583054.1	multicopper oxidase domain-containing protein		499	*Mesorhizobium* sp. YM1C-6-2	90.04	0	870	870	100.00
WP_114350933.1	multicopper oxidase domain-containing protein		493	*Paracoccus lutimaris*	91.43	0	868	868	100.00
WP_122520894.1	multicopper oxidase domain-containing protein		499	*Pannonibacter phragmitetus*	90.04	0	867	867	100.00
